# Myelin Measurement Using Quantitative Magnetic Resonance Imaging: A Correlation Study Comparing Various Imaging Techniques in Patients with Multiple Sclerosis

**DOI:** 10.3390/cells9020393

**Published:** 2020-02-08

**Authors:** Laetitia Saccenti, Akifumi Hagiwara, Christina Andica, Kazumasa Yokoyama, Shohei Fujita, Shimpei Kato, Tomoko Maekawa, Koji Kamagata, Alice Le Berre, Masaaki Hori, Akihiko Wada, Ukihide Tateishi, Nobutaka Hattori, Shigeki Aoki

**Affiliations:** 1 Department of Radiology, Juntendo University School of Medicine, 2-1-1, Hongo, Bunkyo-ku, Tokyo 113-8421, Japan; laetitia.saccenti@gmail.com (L.S.); christina@juntendo.ac.jp (C.A.); sh-fujita@juntendo.ac.jp (S.F.); s.kato.cy@juntendo.ac.jp (S.K.); t-maekawa@juntendo.ac.jp (T.M.); kkamagat@juntendo.ac.jp (K.K.); alice.leberre@ymail.com (A.L.B.); mahori@juntendo.ac.jp (M.H.); a-wada@juntendo.ac.jp (A.W.); saoki@juntendo.ac.jp (S.A.); 2 Department of Radiology, St Joseph Hospital, 185 Rue Raymond Losserand, 75014 Paris, France; 3 Department of Neurology, Juntendo University School of Medicine, 2-1-1, Hongo, Bunkyo-ku, Tokyo 113-8421, Japan; kazumasa@juntendo.ac.jp (K.Y.); nhattori@juntendo.ac.jp (N.H.); 4 Department of Radiology, The University of Tokyo Graduate School of Medicine, 7-3-1, Hongo, Bunkyo-ku, Tokyo 113-0033, Japan; 5 Department of Radiology, Toho University Omori Medical Center, 6-11-1, Omorinishi, Ota-ku, Tokyo 143-8540, Japan; 6 Department of Diagnostic Radiology and Nuclear Medicine, Tokyo Medical and Dental University, 1-5-45, Yushima, Bunkyo-ku, Tokyo 113-8519, Japan; ttisdrnm@tmd.ac.jp

**Keywords:** myelin, multiple sclerosis, synthetic magnetic resonance imaging

## Abstract

Evaluation of myelin by magnetic resonance imaging (MRI) is a difficult challenge, but holds promise in demyelinating diseases, such as multiple sclerosis (MS). Although multiple techniques have been developed, no gold standard has been established. This study aims to evaluate the correlation between synthetic MRI myelin volume fraction (SyMRI_MVF_) and myelin fraction estimated by other techniques, i.e., magnetization transfer saturation (MTsat), T1-weighted images divided by T2-weighted images (T1w/T2w), and radial diffusivity (RD) in patients with MS. We also compared the sensitivities of these techniques for detecting MS-related myelin damage. SyMRI_MVF_, MTsat, T1w/T2w, and RD were averaged on plaque, periplaque white matter, and normal-appearing white matter (NAWM). Pairwise correlation was calculated using Spearman’s correlation analysis. For all segmented regions, strong correlations were found between SyMRI_MVF_ and T1w/T2w (Rho = 0.89), MTsat (Rho = 0.82), or RD (Rho = −0.75). For each technique, the average estimated myelin differed significantly among regions, but the percentage change of NAWM from both periplaque white matter and plaque were highest in SyMRI_MVF_. SyMRI_MVF_ might be suitable for myelin evaluation in MS patients, with relevant results as compared to other well-studied techniques. Moreover, it presented better sensitivity for the detection of the difference between plaque or periplaque white matter and NAWM.

## 1. Introduction

Myelin is important in the transmission of neural information. It maintains the integrity of neural fibers and enhances the speed of propagation of action potentials, which are both essential for proper brain function [1,2]. Various magnetic resonance (MR) techniques have been used to study the pathological evolution of demyelinating diseases, such as multiple sclerosis (MS) [3]. Myelin imaging can potentially play an important role in the clinic, for predicting and monitoring responses to treatment: myelin status has been shown to be associated with changes in functional mobility following therapeutic exercises in patients with MS [4]. However, myelin assessment is a difficult challenge, as it cannot be seen directly on MRI, which has a millimeter-scale resolution. There is currently no recognized gold standard for myelin estimation, although myelin water fraction is one of the best validated and most commonly used quantitative measures for noninvasive assessment of myelin content in the brain [5]. To validate a new myelin estimation technique, histological studies are powerful and considered as the standard criterion, but its significance is somewhat limited as histological staining is evaluated based on optical density contrasts and therefore does not directly quantify myelin [6].

Recently, myelin measurement using quantitative synthetic MRI (SyMRI) was proposed [7]. This approach was originally developed for the simultaneous measurement of longitudinal T1 relaxation rate (R1), transverse T2 relaxation rate (R2), proton density (PD), and local radiofrequency field B1, using multislice, multiecho, and multidelay acquisition. The estimated B1 field is used for the correction of local variations in flip angle. Full head coverage is obtained in approximately 6 minutes [7]. From these absolute parameters, it is possible to create any contrast-weighted image that is clinically useful, including T1-weighted (T1w) or T2-weighted (T2w) images, using SyMRI software [8,9,10]. Using the same absolute parameters, SyMRI also allows myelin measurement [7]. Brain segmentation is based on pre-defined tissue characteristics. The myelin model assumes four compartments in the brain: myelin volume fraction (MVF), cellular volume fraction, free water fraction and excess parenchymal water volume fraction. The model postulates that each compartment has its own R1, R2, and PD, which contribute to the effective R1, R2, and PD of a specific voxel while exchanging magnetization with other partial volume compartments [7]. The MVF measured by SyMRI (SyMRI_MVF_) has been shown to correlate well with histological specimens in nonpathological brains [11] and brains with MS [12] and other myelin measurement methods in healthy subjects [13] and to show good repeatability [14,15] and reproducibility across different in-plane resolutions [16] and scanners [17]. Its clinical utility has been investigated in diseases such as MS [18,19], Sturge–Weber syndrome [20,21], and CADASIL [22].

A widely used myelin imaging method is based on magnetization transfer (MT), which is a physical process by which macromolecules and their closely associated water molecules cross-relax with protons in the free water pool. Based on this phenomenon, it is possible to quantify the protons bound to large molecules, which are not directly visible in MR images due to their extremely short T2. Radiofrequency pulse, applied at an offset to the resonance frequency of water, will cause saturation of the protons in the bound pool, but not those in free water. While returning to equilibrium, macromolecules exchange magnetization with free water, creating a measurable attenuation of the water signal. Myelin estimation using this technique is based on the assumption that most of the macromolecular content in the central nervous system forms part of myelin. Based on this theory, the MT ratio (MTR) has been widely used and has been shown to correlate well with histological myelin content [23]; moreover, an inverse correlation between clinical disability and average lesion MTR has also been reported [24]. However, MTR also correlates with R1, and thus water content, in the MS brain, indicating that inflammation and edema also influence MTR [25,26]. MT saturation (MTsat) imaging was developed to improve the MTR by decoupling the MTR from R1 [27]. MTsat shows higher contrast than MTR in the brain [27] and has been shown to correlate better than MTR with disability metrics in patients with MS [28]. On the other hand, quantitative MT imaging is time-consuming and its post-processing remains challenging.

The T1w/T2w ratio map, which is calculated by dividing T1w images by T2w images, is another approach for assessing tissue microstructure and indirectly, myelin content. T1 is shorter if the water environment contains more microstructural features, such as myelin and macromolecules. T2 is shorter if spins are in a geometrically restricted environment. Thus, the ratio of T1w/T2w images is assumed to accentuate the intrinsic contrast of myelin. Although the T1w/T2w ratio is not a direct index of myelin, it is nevertheless considered a surrogate marker of myelin content [29]. This method was initially developed to evaluate intra-cortical myelin [29,30,31], and myelination of white matter in neonatal brains has also been investigated using this method [32]. The test–retest reliability of the T1w/T2w ratio has been reported to be high [33] and a histological study of patients with MS has shown that the T1w/T2w ratio in the cortex differed significantly between early-stage MS and healthy controls [34]. The major advantage of this method is the use of common T1w and T2w images that have already been acquired in the clinical routine. The T1w/T2w map provides an interesting assessment of tissue microstructure, despite not being highly specific to myelin [33].

On the other hand, diffusion tensor imaging, by measuring the microscopic movement of water in tissues, provides information on central nervous system tissue integrity and structure. Radial diffusivity (RD) provides information about water movement perpendicular to axonal tracts and is known to be related more to myelin integrity than axonal integrity [35]. In the corpus callosum of mouse brains, Song et al. [36] showed that the extent of increased RD reflects the severity of demyelination in the cuprizone demyelination mouse model, while RD decreases with the progression of remyelination. A pathological study in MS subjects concluded that increased RD correlates with demyelination, but also with axon injury [37]. However, diffusion tensor imaging is known to be affected not only by myelin density but also by fiber coherence [38].

A previous study by Hagiwara et al. [13] evaluated the correlation between SyMRI_MVF_, MTsat, and T1w/T2w in healthy controls. There was a strong correlation in the WM between SyMRI_MVF_ and MTsat, indicating that both methods are similarly suitable for measuring myelin in the WM. Although MTsat, T1w/T2w, and RD are considered valid approaches for assessing microstructural integrity and hence myelin status, the relationship between these metrics has been largely unexplored [39]. Furthermore, the correlation between these techniques and a relatively new measure, SyMRI_MVF_, in MS patients has not been evaluated to date.

Correlation studies can reinforce the validity of a new myelin measurement technique if good correlations are found with well-studied methods. Therefore, the aim of our study was to evaluate the correlation between different methods used for myelin evaluation in patients with MS. We focused on four representative myelin imaging techniques, namely, SyMRI_MVF_, MTsat, the T1w/T2w ratio, and RD. We also compared the sensitivities of these techniques for detecting MS-related myelin damage.

## 2. Materials and Methods 

### 2.1. Study Participants

Thirty-seven patients with relapsing-remitting MS were prospectively included in this study, from March 2017 to July 2018. These patients were diagnosed with MS according to the standard McDonald criteria [40]. Of these patients, 14 did not have any plaque or had only small (<5 mm maximal length) plaques on brain MRI and 2 had diffuse extensive WM abnormalities. Therefore, these 16 patients were excluded and the remaining 21 patients were included in the analysis. Disability was assessed using the Expanded Disability Status Scale (EDSS) score. The demographic and clinical data of the patients with MS are provided in Table 1. The institutional review board of Juntendo University Hospital approved this study on 18 March 2016 and written informed consent was obtained from all participants. The ethical approval number is 15-212.

### 2.2. MRI Acquisition Protocol

A 3-T MR system (MAGNETOM Prisma, Siemens Healthcare, Erlangen, Germany) with a 64-channel head coil was used for all imaging. All patients underwent MR relaxometry, MTsat determination, and diffusion-weighted imaging. 

### 2.3. Acquisition and Processing of SyMRI Data

MR relaxometry was performed with the QRAPMASTER (quantification of relaxation times and proton density by multi-echo acquisition of a saturation recovery using turbo spin-echo readout) pulse sequence, which is a multi-slice, multi-echo, and multi-saturation delay acquisition sequence. Two sets of echo times (TE) and 4 sets of delay times were used to generate 8 complex images in each section in order to quantify R1, R2, and PD. The TE were 22 and 99 ms, the delay times were 170, 620, 1970, and 4220 ms and the repetition time (TR) was 4250 ms. An in-plane resolution of 0.8 × 0.8 mm with a slice thickness/gap of 4.0/1.0 mm was used for 30 slices. The field-of-view (FOV) was set to 230 × 186 mm with a matrix size of 320 × 208. The acquisition time was 5 min and 8 s. 

With the assumption that all the R1, R2, and PD values of MVF, excess parenchymal water volume fraction, cellular volume fraction, and free water volume fraction contribute to the effective R1, R2, and PD in each acquisition voxel; a model was produced to estimate partial volumes of these four compartments, as described by Warntjes et al. [7]. This was done by running Bloch equations and optimizing model parameters in a spatially normalized and averaged brain from a group of healthy controls. Damage to myelin, even in the NAWM of patients with MS [18], is supposed to cause deviation from the R1, R2, and PD in the healthy state, leading to a decrease in the calculated SyMRI_MVF_. Using this model, MVF maps were created from R1, R2, and PD maps via SyMRI software (version 8.04; SyntheticMR, Linköping, Sweden).

Even though other myelin imaging techniques require scaling factors for estimating MVF from the measured macromolecular pool or myelin water, this procedure was not necessary because SyMRI_MVF_ directly estimates the volume fraction of myelin in each voxel [7].

### 2.4. Processing of the T1w/T2w Ratio

Synthetic T1w and T2w images were created using post-processing TR of 500 ms and TE of 10 ms and TR of 4500 ms and TE of 100 ms, respectively, on SyMRI software based on R1, R2, and PD maps. To reduce the effect of intensity scaling, we used a calibration algorithm based on 2 anatomical masks (eye and temporal muscle), as proposed by Ganzetti et al. [41]. We recorded the modes as reference values for the eyes as follows: 28.2 for T1w images and 99.9 for T2w images. For the temporal muscle, these values were 58.6 for T1w images and 21.1 for T2w images. After calibrating the T1w and T2w images, their ratio was calculated to produce T1w/T2w ratio images. The T1w/T2w ratio was further linearly calibrated, as reported in a previous study [13], to be comparable to SyMRI_MVF_.

### 2.5. Acquisition and Processing of MTsat

Three three-dimensional (3D) multi-echo fast low-angle shot (FLASH) sequences were run with predominant T1-, PD-, and MT-weighting for all subjects. For T1w images, TR/excitation flip angle α = 10 ms/13° was used; for PD- and MT-weighted images, 24 ms/4° was used. For MT-weighted images, excitation was preceded by an off-resonance Gaussian-shaped RF pulse (frequency offset from water resonance, 1.2 kHz; pulse duration, 9.984 ms; and nominal flip angle, 500°). Other imaging parameters were: slice thickness, 1.8 mm; 104 slices; FOV 224 × 224 mm; matrix 128 × 128, parallel imaging using GRAPPA factor 2 in the phase-encoding direction; 7/8 partial Fourier acquisition in the partition direction; bandwidth 260 Hz/pixel; and total acquisition time of 6 min and 25 s. These 3 images were used to calculate the MTsat index, as described by Helms et al. [27]. The MTsat index was also further linearly calibrated as reported previously [13]. 

### 2.6. Acquisition and Processing of Radial Diffusivity

Whole-brain diffusion-weighted imaging was performed using spin-echo planar imaging employing a *b*-value of 1000 s/mm^2^ along 64 uniformly distributed motion-probing directions in the anteroposterior phase-encoding direction. Standard and reverse phase-encoded blipped images with no diffusion weighting (*b* = 0 s/mm^2^) were also acquired. Other imaging parameters were TR, 3300 ms; TE, 70 ms; flip angle, 90°; FOV, 229 × 229 mm; matrix size, 130 × 130; resolution, 1.8 × 1.8 mm; slice thickness, 1.6 mm; acquisition time, 3 min and 55 s. All datasets were free from severe artifacts, such as gross geometric distortion, signal dropout, or bulk motion. Diffusion-weighted imaging data were then corrected for susceptibility-induced geometric distortions, eddy current distortions, and inter-volume subject motion using TOPUP and EDDY tools [42]. A single-tensor RD map was generated using the DTIFIT tool on FSL software v5.0.11. Linear transformation was performed to register the acquired diffusion and MTsat images to SyMRI images using BBR and FLIRT, respectively, implemented in FSL. 

### 2.7. Image Analysis

**Figure 1:**
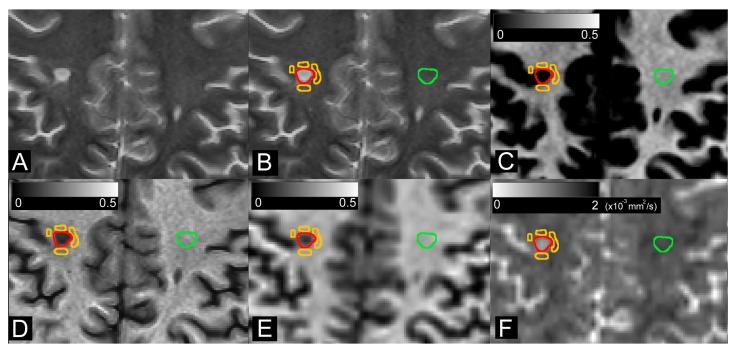
Synthetic T2-weighted images (**A**) were used for semi-automated segmentation of plaque (red), periplaque (orange), and contralateral normal-appearing white matter (green) (**B**). Regions-of-interest were copied and pasted onto SyMRI_MVF_ (**C**), T1w/T2w (**D**), MTsat (**E**), and RD (**F**) maps.

Synthetic T2w images and maps of SyMRI_MVF_, MTsat, RD, and T1w/T2w (Figure 1) were analyzed by using ITK-SNAP software (3.8.0-beta version) by a radiologist (L.S.) with 3 years of experience. Plaques were defined as a WM area of more than 5 mm in diameter in the supratentorial area, with abnormally high intensity on T2w images. Plaques were semi-automatically segmented on synthetic T2w images using an adaptive brush tool for automatic and objective adjustment to lesion boundaries, choosing an adaptive algorithm with high granularity and low smoothness to undersegment plaques slightly in order to mitigate possible misregistration and the partial volume effect. Periplaque regions were manually segmented with a small round-shaped brush (approximately ⅕th of plaque diameter). Periplaque was defined as a visually normal-appearing WM (NAWM) area on a synthetic T2w image closest to a plaque [19,43,44]. Around each plaque, up to 6 areas were added in order to encircle the plaque and, together, these were considered as a periplaque region-of-interest (ROI). NAWM was manually segmented with a round-shaped brush on the WM contralateral to each plaque while avoiding other plaques. All the ROIs placed on synthetic T2w images were copied and pasted onto the SyMRI_MVF_, T1w/T2w, MTsat, and RD maps. 

### 2.8. Statistical Analysis

For acquired myelin-sensitive metrics, normality was tested with the Shapiro–Wilk test. Because not all data were normally distributed, we used the Steel–Dwass nonparametric test for multiple comparisons to compare ROI volumes and the values of SyMRI_MVF_, T1w/T2w, MTsat, and RD among plaque, periplaque, and NAWM tissue. The percentage changes in plaque or periplaque relative to NAWM were also calculated and compared among different metrics using the Steel–Dwass test. The signs of negative percentage changes were inverted before comparison. 

Spearman’s rank order correlation analysis was used to investigate the correlation among myelin-sensitive metrics for plaque, periplaque, NAWM, and all regions. The signs of negative coefficients were inverted for further analysis and classification. Correlation coefficients across metrics were compared in plaque, periplaque, NAWM, and overall regions using a percentile bootstrap method, as described by Wilcox [45,46]. EDSS and disease duration were also correlated with myelin-sensitive metrics in the plaque, periplaque, and NAWM regions, averaged in each patient via Spearman’s rank order correlation analysis. Then, *p*-values for correlation coefficients and their comparisons were controlled for the false discovery rate [47]. Spearman’s ρ correlation coefficients were classified as follows: 0–0.30, very weak; 0.30–0.50, weak; 0.50–0.70, moderate; 0.70–0.90, strong; and 0.90–1.00, very strong. A 2-sided *p*-value <.05 was considered significant. Shapiro–Wilk and Steel–Dwass tests were performed with the software package R, version 3.3.3. Calculation of correlation coefficients and comparisons of these were performed on Matlab (release R2015b, MathWorks, Natick, Massachusetts, United States). 

## 3. Results

Ninety-two isolated plaques were segmented; thus, the total number of ROIs was 276, including plaque, periplaque, and NAWM. The median number of plaques per patient was 5 (range, 1–7). The mean (± standard deviation) size of plaque, periplaque, and NAWM ROIs were 144 ± 111, 144 ± 112, and 145 ± 113 mm^3^, respectively. There were no significant differences between the volumes of these ROIs (*p* > 0.99). 

**Figure 2:**
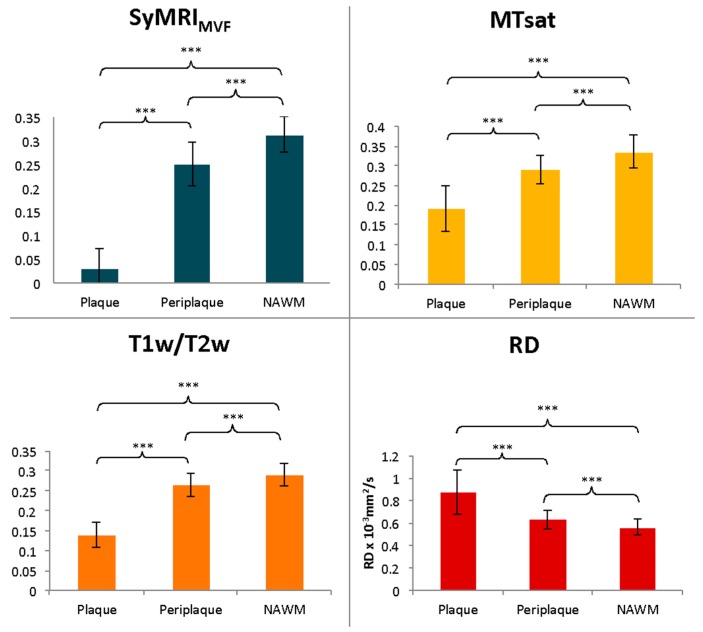
Estimation of myelin with 4 different techniques (SyMRI_MVF_, MTsat, T1w/T2w, and RD). Each technique can differentiate plaque, periplaque, and NAWM regions. These metrics were the lowest in plaque for SyMRI_MVF_, MTsat, and T1w/T2w and the highest in plaque for RD. *** *p* < 0.001.

**Figure 3:**
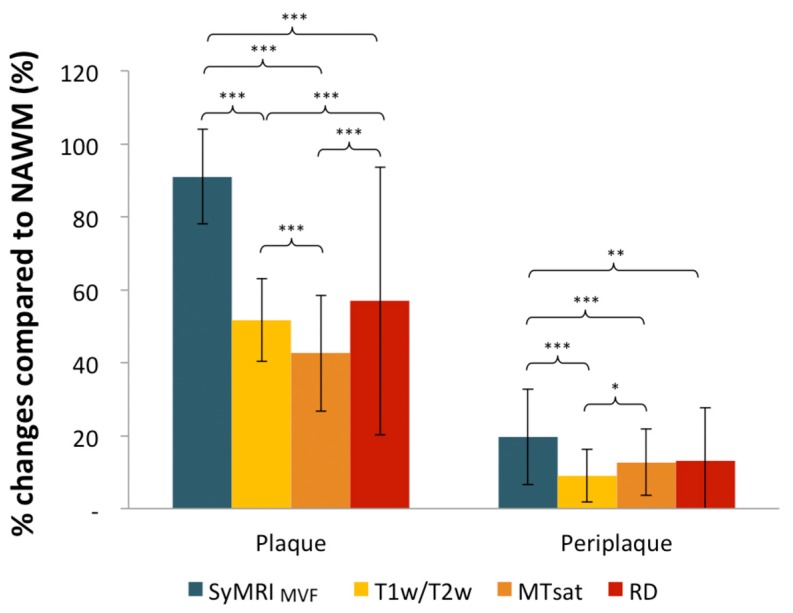
Percentage changes in the metrics (SyMRI_MVF_, T1w/T2w, MTsat, and RD) for plaque and periplaque compared to NAWM regions. SyMRI_MVF_ showed the highest contrast between NAWM and both plaque and periplaque regions. * *p* < 0.05. ** *p* < 0.01. *** *p* < 0.001.

For each myelin measurement technique, metrics were significantly different among plaque, periplaque, and NAWM regions (Figure 2), while the percentage changes between NAWM and both periplaque and plaque regions were significantly the highest for SyMRI_MVF_ (Figure 3). 

All segmented regions together, there were strong significant correlations between SyMRI_MVF_, T1w/T2w, MTsat, and RD, except between RD and T1w/T2w which presented moderate significant correlation (Table 2). The correlation coefficient between SyMRI_MVF_ and T1w/T2w (Rho = 0.89) was significantly higher than all other correlations (Table S1). Correlation coefficients involving RD were significantly lower than other correlations. 

**Figure 4:**
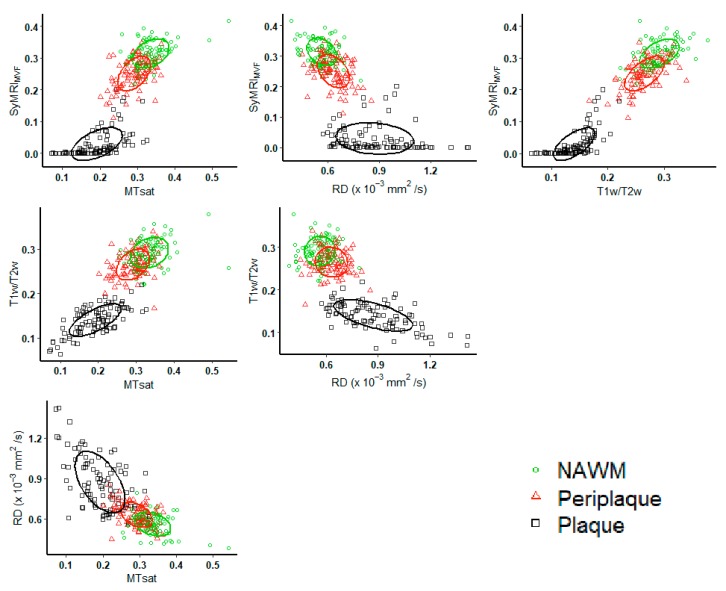
Scatterplots of SyMRI_MVF_, MTsat, T1w/T2w, and RD, plotted by subgroup of segmented regions-of-interest: black square = plaque, red triangle = periplaque, green circle = NAWM. Plot concentration ellipses are also drawn, with concentration levels of 0.5.

We also evaluated the correlation in the subgroups of segmented ROIs (Figure 4, Table 3). Moderate to strong, statistically significant correlations between SyMRI_MVF_, MTsat, and T1w/T2w were found in plaque subgroup, while correlations between RD and other metrics were weak to moderate. Weak to moderate statistically significant correlations between all metrics were found in the periplaque subgroup, except between T1w/T2w and RD, which were nonsignificant. Very weak to weak significant correlations between SyMRI_MVF_, T1w/T2w, and MTsat were detected in NAWM regions. There was no significant correlation between RD and other metrics in the NAWM subgroup. Overall, stronger correlations were found in the plaque subgroup than in the periplaque and NAWM subgroups. Furthermore, correlation coefficients involving RD were lower than other correlations in subgroup analysis (Table S1). 

Disease duration was significantly correlated with SyMRI_MVF_ of NAWM regions averaged in each patient (Rho = −0.63, *p* = 0.002) and with RD of NAWM regions averaged in each patient (Rho = 0.51, *p* = 0.02; Table 4). On the other hand, no significant correlation was found between SyMRI_MVF_ or RD and EDSS, and no significant correlation was found between NAWM MTsat or T1w/T2w and EDSS or disease duration. Likewise, no significant correlation was found between myelin estimation in plaque or periplaque and EDSS or disease duration, regardless of the myelin estimation technique used. 

## 4. Discussion

In this study, we compared four techniques for measuring myelin in the WM of patients with MS. Among all segmented ROIs, we found strong correlations between SyMRI_MVF_ and the other measures. Furthermore, in our study, the contrast for distinguishing plaque and periplaque from NAWM regions was higher with SyMRI_MVF_ than with the other myelin imaging techniques. Because myelin debris is assumed to have much lower R2 than the tightly packed myelin, the contribution of myelin debris to SyMRI_MVF_ is expected to be small. Other myelin imaging techniques may be sensitive to myelin debris or other molecules, which may have resulted in the observed lower contrast between plaque or periplaque and NAWM. Notably, gray matter to WM contrast in the SyMRI_MVF_ (see Figure 1) was reported to be higher than MTsat and T1w/T2w in a previous study by Hagiwara et al. [13] and they concluded that the gray matter to WM contrast in SyMRI_MVF_ was nearer to the results of previous histological studies compared with MTsat and T1w/T2w. Their results also indicate that MTsat and T1w/T2w may be sensitive to other molecules than myelin.

Our results are also in line with those of other previous studies. Hagiwara et al. [43] obtained better contrast using SyMRI_MVF_ than R1, R2, and PD in the WM of MS patients. The MS disease process extends beyond the borders of visible plaques on conventional T2w images, and periplaque and NAWM abnormalities are related to different histological processes, according to pathology studies [44]. Periplaque may be abnormal because of Wallerian degeneration and retrograde degeneration of the cell body [48]. Additionally, plaque evolution (i.e., the demyelination/remyelination process) may explain the difference in myelin measurements in the periplaque regions. In NAWM, microglial activation and axonal degeneration might be the main pathological underpinnings of subtle MRI abnormalities [49]. 

Because the SyMRI model defines myelin at predetermined R1, R2, and PD, based on a group of healthy control subjects [7], any disease process that changes myelin integrity, such as edema or partial demyelination, should theoretically affect SyMRI_MVF_. Hence, SyMRI_MVF_ may be merely a potential additional metric to be confronted with others, such as diffusion tensor imaging and MT imaging. The higher contrast between plaque, periplaque, and NAWM regions seen with SyMRI_MVF_ than with other metrics may indicate that SyMRI_MVF_ has better sensitivity to myelin than other techniques. The major advantage of SyMRI_MVF_ is that the myelin estimation is fully automated and rapid (post-processing time of less than 1 min) and the same 6-minute unique sequence acquisition makes it possible to obtain conventional contrast-weighted images used routinely in the clinic (T1w, T2w, and FLAIR) and other contrast-weighted images (double inversion recovery, phase-sensitive recovery images), with better contrast for MS plaques detection than found with conventional images [10]. 

However, subgroup analyses in this study found lower correlations between the techniques, especially in NAWM regions. Calculation of correlation coefficients depends largely on the range of the measured values: if the range is wide, the correlation will be higher than if the range is narrow [50]. Hence, it is natural that the correlation coefficient was lowest when calculated for only NAWM, which is expected to be homogeneous, as compared with pathological plaque and periplaque regions. These results were similar to those of previous myelin correlation studies: correlation between the myelin water fraction and MTR was very weak within NAWM in MS patients [51]. Another correlation study analyzed the relationship between RD, MTR, and the myelin water fraction and found good agreement when estimating WM microstructural damage in MS patients, with the lowest correlations found in the NAWM [39]. Recently, O’Muircheartaigh et al. [52] also showed strong correlations throughout the brain between the myelin water fraction, MTR, and quantitative T1, but these relationships varied in different tissue types. In NAWM, no significant correlation was found. All these studies concluded that the different quantitative techniques are at least partially correlated with each other but are sensitive to different aspects of the pathology and may provide complementary information about underlying brain tissues [53].

Although promising advances in imaging myelination have been reported, inflammation can interfere with measuring myelin and axons. NAWM in MS patients often exhibits chronic injury, microglial activation, gliosis, and increased expression of proteolytic enzymes [54]. Moll et al. [49], in a pathology-imaging correlation study, concluded that the pathological substrates of MTR changes in NAWM in MS patients could be attributed to axonal degeneration and microglial activation, but were not correlated to myelin staining. 

Even though the specificity of myelin quantification by SyMRI_MVF_ was not proven in this study, SyMRI_MVF_ averaged for the NAWM in each patient presented a significant correlation with disease duration. This emphasizes the clinical relevance of NAWM and the importance of detecting damage to this tissue, considering that the association between clinical findings and lesion volumes is reportedly poor [55].

No significant correlation was found between EDSS and the estimated myelin in the NAWM, but the range of EDSS in this study (i.e., 0–2) was small. Further studies that include a larger population of patients with a wider range of disability scores are warranted. 

Our study had some limitations. First, we did not compare SyMRI to myelin water imaging, which is one of the best validated techniques. Further studies comparing SyMRI_MVF_ and myelin water imaging in patients with MS should be conducted to analyze the association between these metrics. Second, we chose to investigate MTsat, which is thought to be an improvement over MTR, but some new magnetization transfer techniques, for example, inhomogeneous magnetization transfer, have been developed that may increase the sensitivity and specificity for myelin [56]. Third, even though we slightly undersegmented plaques to mitigate possible misregistration and the partial volume effect, the acquired images were gapped at 1 mm and there may have been inevitable partial volume effects that have contributed to bias in the results. Fourth, we focused on T2w WM hyperintense lesional tissue without taking the T1w signal and contrast-enhancement, which requires the administration of a gadolinium-based contrast agent, into account when examining the activity of lesions. In the future, contrast-enhanced images could be used to separate subgroups or inflammatory lesions and study the differences in the metrics. A previous study by Blystad et al. [57] compared T1, T2, and PD measured by SyMRI between enhancing and NAWM and showed that enhancing lesions have higher T1, T2, and PD values than NAWM. Even though there has been no study that has evaluated the difference in SyMRI_MVF_ between enhancing lesions and NAWM, it is highly likely that enhancing lesions show decreased SyMRI_MVF_ than NAWM. However, to what degree inflammation contributes to SyMRI_MVF_ is still unclear and a future histological study on active MS lesions is awaited. Additionally, the patients included in the present study were all being followed-up for relapsing–remitting MS. In future studies, myelin should also be evaluated in patients with clinically isolated syndromes and primary/secondary progressive MS and the correlations between the techniques assessed. 

Lastly, concerning NAWM, we did not take into account the location of the ROI; hence, future studies should include more patients and, using anatomical masks, evaluate subgroups or anatomical regions in order to establish the influence of ROI location on the metrics. 

## 5. Conclusions

SyMRI_MVF_ might be suitable for myelin evaluation of patients with MS, with results comparable to those of other well-studied myelin evaluation techniques (MTsat, T1w/T2w, and RD). Moreover, it presented better sensitivity for detecting the difference between plaque or periplaque regions and NAWM, which warrants further investigation for SyMRI_MVF_ to be useful for diagnosis and prognosis evaluation. Subregional analysis demonstrated a weaker but significant correlation in myelin estimation, especially in NAWM, but NAWM SyMRI_MVF_ was significantly associated with disease duration. SyMRI_MVF_ could therefore yield additional complementary information about microstructural tissue damage in patients with MS. 

## Figures and Tables

**Table 1:** Demographic and clinical details of study participants.

	MS Patients
**No. of Subjects**	21
**Mean Age (yr)**	37.9 ± 9.9
**Sex (Male/Female)**	2:19
**Disease Duration (Mean) (yr)**	8.7 ± 6.5
**EDSS Score (range)**	1 (0–2)

**Table 2:** Correlation matrix of Spearman’s rank order correlation analysis.

	MTsat	T1w/T2w	RD
**SyMRI_MVF_**	0.82 [0.77–0.87] ***	0.89 [0.85–0.92] ***	−0.75 [−0.80–(−0.69)] ***
**MTsat**		0.80 [0.74–0.85] ***	−0.72 [−0.78–(−0.65)] ***
**T1w/T2w**			−0.66 [−0.73–(−0.57)] ***

Data are the Spearman’s Rho correlation coefficients ± 95% confidence interval. For the segmented regions overall, strong correlations were found between SyMRI_MVF_, T1w/T2w, MTsat, and RD, except for RD and T1w/T2w, which were moderately correlated. All correlations were statistically significant. *** *p* < 0.001.

**Table 3:** Correlation among SyMRI_MVF_, MTsat, T1w/T2w, and RD for plaque, periplaque, and NAWM subgroups.

		MTsat	T1w/T2w	RD
**Plaque**	**SyMRI_MVF_**	0.70 [0.58–0.79] ***	0.78 [0.67–0.86] ***	−0.38 [−0.53–(−0.19)] ***
	**MTsat**		0.64 [0.47–0.77] ***	−0.48 [−0.65–(−0.29)] ***
	**T1w/T2w**			−0.58 [−0.71–(−0.41)] ***
**Periplaque**	**SyMRI_MVF_**	0.45 [0.23–0.60] ***	0.62 [0.49–0.74] ***	0.41 [0.23–0.56] ***
	**MTsat**		0.41 [0.22–0.59] ***	0.40 [0.19–0.59] ***
	**T1w/T2w**			−0.09 [−0.31–0.13]
**NAWM**	**SyMRI_MVF_**	0.33 [0.13–0.51] **	0.50 [0.32–0.67] ***	−0.21 [−0.41–(−0.02)]
	**MTsat**		0.28 [0.11–0.47] *	−0.20 [−0.45–0.02]
	**T1w/T2w**			0.11 [−0.10–0.29]

Data are the Spearman’s Rho correlation coefficients ± 95% confidence interval. * *p* < 0.05, ** *p* < 0.01, *** *p* < 0.001.

**Table 4:** Correlation between clinical scores and SyMRI_MVF_, MTsat, T1w/T2w, or RD in plaque, periplaque, and NAWM.

		SyMRI_MVF_	MTsat	T1w/T2w	RD
**Plaque**	**EDSS**	−0.10 [−0.57 to 0.39]	−0.29 [−0.71 to 0.22]	−0.0014 [−0.49 to 0.48]	−0.12 [−0.56 to 0.36]
	**Disease Duration**	0.20 [−0.36 to 0.64]	0.17 [−0.34 to 0.63]	−0.17 [−0.67 to 0.38]	0.32 [−0.17 to 0.77]
**Periplaque**	**EDSS**	0.13 [−0.33 to 0.51]	0.12 [−0.38 to 0.57]	0.24 [−0.26 to 0.66]	−0.31 [−0.68 to 0.11]
	**Disease Duration**	−0.00065 [−0.52 to 0.45]	−0.12 [−0.51 to 0.38]	−0.24 [−0.67 to 0.26]	0.23 [−0.25 to 0.66]
**NAWM**	**EDSS**	0.45 [0.031 to 0.76]	0.15 [−0.32 to 0.60]	0.23 [−0.28 to 0.63]	−0.47 [−0.80 to −0.056]
	**Disease Duration**	−0.63 [−0.81 to −0.32] **	−0.39 [−0.70 to 0.072]	−0.38 [−0.72 to 0.077]	0.51 [0.11 to 0.81] *

Data are the Spearman’s Rho correlation coefficients ± 95% confidence interval. * *p* < 0.05, ** *p* < 0.01, and *** *p* < 0.001.

## Supplementary Materials

The following are available online at https://www.mdpi.com/2073-4409/9/2/393/s1, Table S1: *p* values for comparisons of Spearman’s rho correlation coefficients across SyMRI_MVF_, MTsat, T1w/T2w and RD. These *p* values are corrected for false discovery rate.
