# Supplementary material for: Myelin Measurement Using Quantitative Magnetic Resonance Imaging: A Correlation Study Comparing Various Imaging Techniques in Patients with Multiple Sclerosis
Source: Cells. 2020 Feb 8;9(2):393. doi: 10.3390/cells9020393 (PMC7072333; doi:10.3390/cells9020393)
Supplement: Supplementary file 1 [file cells-09-00393-s001.pdf]

Supplementary Table 1. P values for comparisons of Spearman's rho correlation coefficients across $SyMRI_{MVF}$, MTsat, T1w/T2w, and RD. These P values are corrected for false discovery rate.

	Spearman's Rho	$SyMRI_{MVF}$ vs. T1w/T2w	$SyMRI_{MVF}$ vs. RD	MTsat vs. T1w/T2w	MTsat vs. RD	T1w/T2w vs. RD
All ROI	$SyMRI_{MVF}$ vs. MTsat	<0.001	0.018	0.11	<0.001	<0.001
	$SyMRI_{MVF}$ vs. T1w/T2w		<0.001	<0.001	<0.001	<0.001
	$SyMRI_{MVF}$ vs. RD			0.13	0.29	<0.001
	MTsat vs. T1w/T2w				0.038	<0.001
	MTsat vs. RD					0.075
Plaque	$SyMRI_{MVF}$ vs. MTsat	0.24	<0.001	0.34	0.060	0.24
	$SyMRI_{MVF}$ vs. T1w/T2w		<0.001	0.067	<0.001	0.036
	$SyMRI_{MVF}$ vs. RD			0.03	0.23	0.067
	MTsat vs. T1w/T2w				0.067	0.46
	Mtsat vs. RD					0.24
Periplaque	$SyMRI_{MVF}$ vs. MTsat	0.19	0.96	0.96	0.96	0.015
	$SyMRI_{MVF}$ vs. T1w/T2w		0.13	0.13	0.13	<0.001
	$SyMRI_{MVF}$ vs. RD			0.96	0.96	<0.001
	MTsat vs. T1w/T2w				0.96	<0.001
	Mtsat vs. RD					0.06
NAWM	$SyMRI_{MVF}$ vs. MTsat	0.47	0.63	0.78	0.63	0.35
	$SyMRI_{MVF}$ vs. T1w/T2w		0.30	0.30	0.30	<0.001
	$SyMRI_{MVF}$ vs. RD			0.77	0.94	0.73
	MTsat vs. T1w/T2w				0.77	0.48
	MTsat vs. RD					0.73
